# LQUnet: a vascular segmentation network based on multi-scale feature fusion and hierarchical self distillation

**DOI:** 10.1038/s41598-026-49862-9

**Published:** 2026-04-27

**Authors:** Pengjin Liu, Keyuan Qiu, Dong Wang, Zixuan Wang, Quan Qi

**Affiliations:** 1 Shihezi University, Xiangyang Street, Shihezi, 832003 Xinjiang China; 2 Qingdao Hospital University of Health and Rehabilitation Sciences, Qingdao Municipal Hospital, JiaoZhou Road, Qingdao, 266000 Shandong China; 3 Qiaoverse Technology Co., Ltd., Suzhou, Jiangsu China

**Keywords:** Hepatic vessel segmentation, Centreline guidance, Deep learning, LQUnet, Gate attention, Multi-scale feature fusion, Computational biology and bioinformatics, Engineering, Mathematics and computing

## Abstract

Vessel segmentation has important clinical significance in medical image analysis, especially in the assessment of liver disease and preoperative planning for the accurate extraction of vascular structures puts forward higher requirements. To cope with the challenges of complex vascular morphology, diverse scales and easy loss of fine vessels, this paper proposes a novel vascular segmentation network– LQUnet, which is based on supervised learning and incorporates a centreline guidance strategy to enhance the attention to the target vascular region while maintaining the ability to model the global contextual information. LQUnet introduces a gated attention mechanism and a multi-scale feature fusion module in the encoder to enhance the feature extraction capability of complex structures, and combines jump connection and branch reconstruction mechanisms in the decoder to achieve detail restoration. In addition, a composite loss function combining cross-entropy, Dice, Focal loss and hierarchical self-distillation is designed to strengthen the model’s ability to learn fine-grained features. Experimental results on several publicly available datasets and self-constructed hepatic vessel datasets show that LQUnet outperforms existing methods in terms of overall segmentation accuracy and structural preservation of fine-grained vessels, and has good potential for clinical applications.

## Introduction

Vessel segmentation is a critical task in medical image processing, playing an important role in cardiovascular disease assessment, tumor vasculature analysis, and surgical navigation^1^. However, the complexity of vascular structures–such as branching diversity and scale variation–along with the low contrast between small vessels and surrounding tissues, makes achieving high-precision automatic segmentation extremely challenging^2,3^.

The rise of deep learning technologies has brought revolutionary progress to the field of medical image segmentation. Convolutional neural networks (CNNs), with their powerful feature extraction capabilities, have dominated a variety of medical image segmentation tasks. Among them, U-Net^4^, with its encoder-decoder structure and skip connections, has demonstrated outstanding segmentation performance and is widely used in vascular segmentation. Inspired by U-Net, numerous improved models have been proposed, such as UNet++^5^, DenseUNet^6^, U-Net v2^7^, FusionU-Net^8^, and SelfReg-UNet^9^. These models enhance segmentation accuracy and generalization capabilities through strategies such as nested connections, regularization mechanisms, or dense connections.

However, the inherent limitations of CNNs hinder further performance breakthroughs. Due to the local receptive field nature of convolutional operations, CNNs struggle to capture global contextual information and long-range dependencies–especially when dealing with tiny vessels and complex branching structures^10^. Although some methods attempt to model long-range dependencies through dilated convolutions^11^ or non-local attention mechanisms^12^, their ability to model global context in vessel segmentation tasks remains insufficient, particularly in low-contrast and lesion-confounding scenarios^13^.

In recent years, the Transformer architecture–originally successful in the natural language processing field–has been introduced to computer vision (CV) tasks. Its multi-head self-attention (MSA) mechanism can effectively model global dependencies, offering a new perspective for overcoming the limitations of CNNs^14^. In medical image segmentation, Vision Transformer (ViT)^15^, Swin Transformer^16^, and Segmentation Transformer (SETR)^17^ have demonstrated strong feature extraction capabilities. For vessel segmentation, TransUNet^18^ fuses CNN and Transformer features to extract multi-scale representations, while TransFuse^19^ further optimizes their synergy. UNETR^20^ and SwinUNETR^21^ bring Transformers into 3D medical image segmentation, significantly enhancing the ability to segment complex vascular structures.

However, despite these advances, the application of Transformer modules in the decoder stage remains underexplored. In particular, the optimization of multi-scale feature extraction for handling diverse vessel thicknesses and complex morphologies is still insufficient. Moreover, in the absence of structural priors, it is difficult to maintain the topological consistency of vessel branches. These limitations have also been highlighted in recent survey studies, which emphasize that current Transformer-based medical segmentation frameworks still face challenges in structural modeling and standardized design^22^.

As an auxiliary strategy for vessel segmentation, centerline guidance has shown promise: Zhao et al.^23^ proposed a centerline-based liver vessel image restoration method, which enhanced the segmentation accuracy of small vessels by providing topological priors. However, its deep integration with Transformers remains to be explored.

Additionally, the Transformer module is computationally expensive and inefficient when directly applied to high-resolution medical images. To address these challenges, some studies have introduced gating mechanisms to improve the expressiveness and computational efficiency of attention modules. For instance, Gated Axial-Attention^20^ and the Breakpoint Attention mechanism in MCG and BA-Net^24^ enable dynamic adjustment of attention weights, thereby enhancing the model’s ability to perceive fine-grained features such as tiny vessels.

Furthermore, recent studies have begun to incorporate frequency-domain modeling into attention-based frameworks to enhance global context representation. Zhang et al. proposed MISATrack^25^, which integrates a frequency-domain encoder based on discrete wavelet transform (DWT) with a scale-frequency linear attention (SFLA) mechanism, enabling efficient multi-frequency feature integration while maintaining real-time performance. Similarly, Zhang et al. introduced GLKA-UNet^26^, which employs fast Fourier transform (FFT) to extract global frequency-domain features and fuses them with spatial-domain representations. In addition, a hierarchical feature enhancement module combined with KAN attention is designed to improve robustness to scale variation and complex background noise. These methods demonstrate that integrating frequency-domain information with attention mechanisms can effectively improve feature representation and global modeling capability.

On the other hand, facing common problems in medical images like class imbalance and detail ambiguity, knowledge distillation (KD)^27^ has been proposed to improve the model’s ability to perceive fine-grained structures. LightVessel^28^ and GKD-Net^29^ have explored the balance between lightweight design and generalization in vessel and cardiac segmentation tasks. Hierarchical distillation further improves structural boundary awareness by guiding student networks to learn feature distributions at different scales or semantic levels from teacher networks^30^.

Nevertheless, recent studies suggest that simply integrating multiple modules (e.g., feature fusion, attention mechanisms, and structural priors) does not necessarily guarantee performance improvement, unless their contributions are carefully designed and systematically validated^31^.

To address the contradiction between insufficient detail recognition and weak structural modeling in vessel segmentation, we propose a novel framework–LQUnet. LQUnet employs ResNetV2 for image feature extraction and performs multi-scale feature fusion using features from different CNN layers. It also integrates centerline guidance to enhance segmentation of small vessels and complex morphologies.

In the encoder, LQUnet introduces a gated attention mechanism, enabling the model to adaptively adjust the weight of each attention head according to the input, thus efficiently integrating global contextual information.

In the decoder, LQUnet not only preserves the global context from the encoder but also incorporates intermediate encoder features as skip connections, further improving the quality of feature recovery during upsampling.

Moreover, we introduce a self-distillation loss, and employ a composite loss function that combines Focal Loss and hierarchical self-distillation loss to enable end-to-end training. This enhances the model’s performance in fine-grained medical image segmentation tasks.

Extensive experiments on multiple vessel segmentation datasets demonstrate that LQUnet performs well in small vessel segmentation, complex morphology handling, and overall accuracy. The main contributions of this paper are as follows:

1. *A centerline-guided progressive vessel segmentation framework is proposed*: By integrating a centerline sampling strategy, the model’s focus on target vascular regions is effectively enhanced, background interference is reduced, and segmentation accuracy and efficiency are improved.
2. *A multi-scale feature fusion and gated attention mechanism is designed*: A dynamic multi-scale feature fusion strategy and gated attention mechanism are introduced in the encoder to significantly enhance the perception of vascular structures.
3. *A hierarchical self-distillation loss and composite loss function are proposed*: By introducing a hierarchical self-distillation technique and combining cross-entropy loss, Dice loss, and Focal loss, the model’s ability to capture fine-grained features is optimized.
4. *The superiority of the method is validated on vessel segmentation tasks*: Experiments on multiple vessel segmentation datasets show that LQUnet outperforms existing methods in small vessel segmentation and complex morphology handling, demonstrating its effectiveness and superiority.

## Releated works

In this section, we first summarize typical U-Net-based methods for vessel segmentation, with a particular focus on the application of the encoder-decoder structure and skip connections in handling vascular structures.

Then, we provide an overview of recent advances in gated attention mechanisms in vessel segmentation, especially their role in enhancing the representation of complex vascular features.

Finally, we review the application of knowledge distillation-based loss function design in vessel segmentation, highlighting its contribution to performance optimization and introducing our proposed improvement strategy.

### U-Net-based vessel segmentation methods

In recent years, incorporating topological optimization priors into convolutional or hybrid networks has become a key trend for improving the accuracy of complex lesion and tissue segmentation^32^. U-Net^4^, known for its encoder-decoder architecture and skip connections, has demonstrated excellent performance in vessel segmentation and has become a preferred method for handling complex vascular structures. Numerous improvements to the traditional U-Net have been proposed. For instance, Zhou et al. developed UNet++^5^, which enhances multi-scale feature fusion through nested skip connections, significantly improving segmentation accuracy for small vessels. Similarly, Li et al.^6^ proposed DenseUNet, which improves segmentation performance in low-contrast vessels by enhancing feature reuse via dense connections. U-Net v2^7^ significantly boosts multi-scale feature fusion by introducing Hadamard product-based skip connections, while FusionU-Net^8^ adopts a dual-path fusion strategy to bridge the semantic gap between encoder and decoder. SelfReg-UNet^9^, on the other hand, introduces a self-regularization mechanism to enhance generalization ability, showing strong stability in small-sample medical image segmentation scenarios. Inspired by these methods, LQUnet adopts a U-shaped architecture and performs multi-scale feature fusion via cascaded upsamplers, achieving precise segmentation of complex vascular structures. Recent studies have also emphasized that the effectiveness of such multi-module architectures depends on well-structured design and rigorous validation, particularly when combining feature fusion and attention mechanisms^31^.

### Applications of gated attention mechanisms in vessel segmentation

In recent years, attention mechanisms have shown remarkable success in medical image segmentation^33–35^, especially in tasks involving complex structures and small targets such as vessels. An increasing number of studies have introduced gated attention mechanisms to dynamically regulate attention flow, thereby improving feature selection precision and enhancing robustness against noise. Medical Transformer (MedT) integrates a Gated Axial Attention module that effectively models long-range dependencies along spatial dimensions while suppressing the propagation of irrelevant features through gating mechanisms, significantly improving performance on cerebral vessel datasets. The Connection Sensitive Attention U-Net (CSAU)^36^ enhances the perception of fine vascular structures by incorporating a connection-sensitive loss function and gated attention modules, achieving excellent performance in retinal vessel segmentation. MCG & BA-Net^24^ combine Multiscale Context Gating and Breakpoint Attention mechanisms to effectively boost recognition and segmentation accuracy of tiny vessels.

These studies suggest that incorporating gating mechanisms can effectively mitigate the dispersion problem of conventional attention in noisy regions and enhance the model’s representation of small vessels and complex branching structures. In addition, recent reviews indicate that attention-based architectures, especially those involving Transformer variants, require careful architectural design and standardized evaluation to ensure robustness and reproducibility in medical imaging tasks^22^. Drawing from these approaches, LQUnet integrates a gated attention mechanism, which dynamically adjusts attention distribution via gating units, further improving the model’s robustness in segmenting complex vascular structures.

In addition to gated attention, dual-attention strategies have shown promise in capturing spatial and channel dependencies, such as the DA-TransUNet architecture^37^. Additionally, multi-scale context aggregation techniques, like the gradient-guided boundary-aware selective scanning^38^, provide robust mechanisms for handling diverse lesion morphologies by adaptively emphasizing relevant spatial regions.

### Applications of knowledge distillation-based loss functions in vessel segmentation

Knowledge distillation has demonstrated potential in performance optimization for vessel segmentation by transferring knowledge from complex models to lightweight ones^39,40^. Hinton et al.^27^ first proposed knowledge distillation, using KL divergence to guide the student model in learning the soft label distribution from the teacher model, thereby improving segmentation accuracy. Building on this, the LightVessel framework^28^ introduced Feature-wise Similarity Distillation (FSD) and Adversarial Similarity Distillation (ASD) modules, significantly improving segmentation performance of coronary arteries. Furthermore, Qi et al. proposed a generalized knowledge distillation framework, designing Dual Contrastive Graph Distillation (DCGD) and Domain-Invariant Cross Distillation (DICD) methods to enhance model performance in retinal vessel segmentation tasks^29^. Zhang et al. introduced the EXP-Net framework, which performed well in weakly connected and low-contrast vascular regions^41^. Araújo et al. proposed a topology similarity loss function based on morphological closing operations^30^, aiming to maintain the topological consistency of vascular structures and reduce disconnections and artifacts. Inspired by these works, LQUnet leverages distillation losses to improve model robustness and generalization ability, thereby achieving superior performance in vessel segmentation tasks. Moreover, recent studies have pointed out that the effectiveness of distillation strategies is closely related to the transparency of training protocols and the consistency of evaluation settings^22^.

## Method

**Fig. 1:**
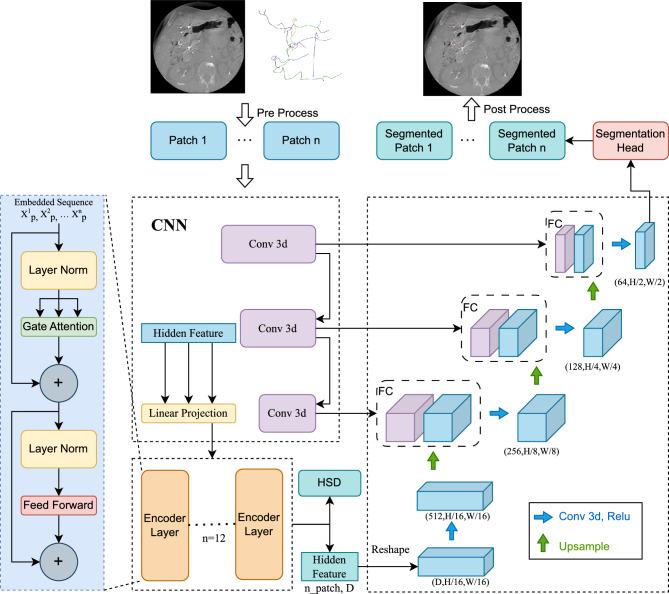
overall structure.

As illustrated in Fig. 1, this study proposes a centerline-guided progressive vessel segmentation framework. The overall method takes a 3D medical image as input. The image first undergoes a preprocessing stage. It is important to note that the patch-wise sampling along the vessel centerline is only performed for liver vessel segmentation; this step is omitted for other datasets.

After preprocessing, the image is sliced axially into 2D slices and subjected to standard operations such as normalization and resizing. The resulting slices are then fed into a convolutional neural network (CNN) backbone to extract multi-level features. To enhance the model’s ability to perceive fine-grained vascular structures, a multi-scale feature fusion module is introduced at the feature extraction stage. This module integrates semantic information from different levels to improve representation capability.

The fused features are then passed into a Transformer architecture, where they undergo layer-by-layer processing through the encoder and decoder to model global contextual dependencies and recover fine details. At the end of the network, the processed features are passed through a segmentation head and a distillation head to generate vessel predictions.

Simultaneously, after the encoder stage, features are passed into a hierarchical self-distillation (HSD) head to compute the distillation loss. Finally, the model is trained in an end-to-end fashion using a combined loss composed of Focal Loss and hierarchical self-distillation loss, which enhances sensitivity to small vessels and improves generalization performance.

### Data preprocessing

To accommodate the requirements of different segmentation tasks, two data preprocessing strategies are designed in this study. Unlike other tasks^42–45^, for conventional medical image segmentation tasks, an axial slicing strategy is applied to process 3D images: the original 3D image $\textbf{S} \in \mathbb {R}^{H \times W \times D}$ is sliced layer by layer into 2D slices, resulting in each 2D slice $\textbf{S}_{i} \in \mathbb {R}^{H \times W}$. These slices are then normalized and resized to ensure consistency for network training and inference.

For the centerline-guided progressive vessel segmentation task, which is the focus of this study, a centerline-based sampling strategy is designed to address the challenges posed by the fine, complex, and sparsely distributed nature of vascular structures. Let the vessel centerline be denoted as $\mathcal {L} = { \textbf{l}_1, \textbf{l}_2, \dots , \textbf{l}_i, \dots , \textbf{l}_N }$, where $\textbf{l}_i$ represents the *i*-th point on the centerline. To extract image patches surrounding the vessel regions, local sampling is performed in the original image based on the coordinates of the centerline.

Specifically, we define a fixed window region $\mathcal {R}_i$, centered at the *i*-th centerline point $\textbf{l}_i$, with a sampling radius of $\Delta$. Through this progressive sampling approach, image patches can be extracted around vessel regions, which effectively enhances the model’s focus on target vessels, reduces background interference, and provides clearer structural inputs for subsequent feature extraction and segmentation.

The sampling region $\mathcal {R}_i$ is defined as:

1 $$\begin{aligned} \mathcal {R}_i = \left\{ \textbf{I}_{x, y, z} \,\big |\, \textbf{l}_i - \Delta \le \textbf{I}_{x, y, z} \le \textbf{l}_i + \Delta \right\} \end{aligned}$$

### Encoder

In this study, we designed an encoder that combines multi-scale feature fusion with a gated self-attention mechanism to enhance feature representation for vascular segmentation. The encoder consists of two core modules: 1. Multi-scale Feature Fusion based on CNN and 2. Gated Self-Attention Mechanism. Each module is described in detail below.

#### Multi-scale feature fusion based on ResNetV2

We use ResNetV2 to extract multi-scale features and fuse them via a FeatureFusion module. Let the input image have size $H_{\text {in}} \times W_{\text {in}}$, e.g., $256\times 256$, with 3 channels. The network extracts three layers of features $\{F_1, F_2, F_3\}$ with channel numbers and spatial resolutions as follows:

- $F_1 \in \mathbb {R}^{C_1 \times H_1 \times W_1}$, $C_1 = 64$, $H_1 = W_1 = H_{\text {in}}/2$, capturing low-level local texture information;
- $F_2 \in \mathbb {R}^{C_2 \times H_2 \times W_2}$, $C_2 = 256$, $H_2 = W_2 = H_{\text {in}}/4$, containing mid-level contextual features;
- $F_3 \in \mathbb {R}^{C_3 \times H_3 \times W_3}$, $C_3 = 512$, $H_3 = W_3 = H_{\text {in}}/8$, carrying high-level global information.

The three feature layers are fused as follows:

- First-layer feature: keep unchanged, i.e., $F_1$;
- Second-layer fusion:
  2 $$\begin{aligned} F_2' = \text {Upsample}_{\text {bilinear}}\Big (\text {Conv}_{1\times 1}(F_1, C_2)\Big ) + F_2 \end{aligned}$$
  where the $1\times 1$ convolution maps $F_1$ from $C_1 \rightarrow C_2$, and bilinear upsampling aligns the spatial size $H_1 \times W_1$ to $H_2 \times W_2$;
- Third-layer fusion:
  3 $$\begin{aligned} F_3' = \text {Upsample}_{\text {bilinear}}\big (\text {Conv}_{1\times 1}(F_1, C_3)\big ) + \text {Upsample}_{\text {bilinear}}\big (\text {Conv}_{1\times 1}(F_2, C_3)\big ) + F_3 \end{aligned}$$
  where $1\times 1$ convolutions map $F_1$ and $F_2$ channels to $C_3$, and upsampling aligns spatial size to $H_3 \times W_3$.

The final fused feature set $\{F_1, F_2', F_3'\}$ is used as input for the downstream vascular segmentation module. Dynamic $1\times 1$ convolutions and upsampling ensure channel matching and spatial alignment, allowing features from different scales to be directly added. Features are reversed according to ResNetV2 output order before being passed to subsequent modules to maintain bottom-up multi-scale fusion.

The design rationale behind this multi-scale feature fusion module differs fundamentally from standard U-Net skip connections and typical pyramid-based fusion strategies (e.g., FPN). Standard skip connections bypass the deepest layers and concatenate features exclusively in the decoder, which leaves the bottleneck block–our Transformer encoder–blind to high-resolution local textures. On the other hand, conventional pyramid strategies typically employ a top-down semantic pathway.In contrast, our module utilizes a bottom-up additive strategy strictly within the encoder. Tiny vessels are extremely fragile and easily lost after consecutive pooling operations. By dynamically aligning and explicitly injecting high-resolution spatial details (e.g., $F_1$) into deeper semantic representations ($F_2$ and $F_3$) before sequence embedding, we ensure that the fragile structural priors survive. Consequently, the subsequent Transformer can simultaneously model global contextual dependencies and delicate vascular topologies, effectively preventing the degradation of fine-grained features.

#### Gated self-attention mechanism

Standard multi-head self-attention (MSA) often suffers from ”head dispersion” in noisy medical scans, where attention weights are erroneously distributed across non-vascular background regions. Unlike existing gated approaches such as Gated Axial-Attention which primarily focus on spatial axis reduction, our Gated Attention (GA) functions as a dynamic ”head filter” within the Transformer encoder. By assigning learnable weights *G* to each individual head, the model can adaptively suppress entire heads that capture noise and emphasize those representing critical vascular topologies. This head-wise regulation is essential for maintaining the continuity of thin vessels in low-contrast environments.

In the Transformer encoder, we introduce a gated attention mechanism to adaptively regulate the importance of different attention heads. Standard self-attention computes the attention matrix $A = \text {Softmax}(QK^\top / \sqrt{d_k})$ to capture relationships among elements, but it cannot distinguish the contribution of each attention head. To address this, we design a gating module that assigns dynamic weights to each head, enhancing the model’s expressive power.

Given the input hidden state $\textbf{H} \in \mathbb {R}^{B \times N \times C}$, where *B* is batch size, *N* is sequence length, and *C* is hidden dimension, the query, key, and value matrices are $\textbf{Q}, \textbf{K}, \textbf{V} \in \mathbb {R}^{B \times h \times N \times d_h}$, with *h* attention heads and $d_h = C / h$ dimension per head. Standard multi-head attention computes:

4 $$\begin{aligned} A = \text {Softmax}\Big (\frac{\textbf{Q} \textbf{K}^\top }{\sqrt{d_h}}\Big ) \in \mathbb {R}^{B \times h \times N \times N} \end{aligned}$$

The gating module generates a weight for each head via a small feedforward network $\mathcal {G}$:

5 $$\begin{aligned} \textbf{G} = \sigma (\mathcal {G}(\textbf{H})) \in \mathbb {R}^{B \times h \times N \times 1} \end{aligned}$$

where $\sigma$ is the Sigmoid function, constraining values to [0, 1]. Each attention head is scaled:

6 $$\begin{aligned} A' = A \odot \textbf{G}_{\text {expanded}} \in \mathbb {R}^{B \times h \times N \times N} \end{aligned}$$

where $\textbf{G}_{\text {expanded}}$ is broadcast along the last dimension to match *A*. The final context representation is:

7 $$\begin{aligned} \textbf{O} = A' \textbf{V} \in \mathbb {R}^{B \times h \times N \times d_h} \end{aligned}$$

All heads are then concatenated and linearly projected back to hidden dimension *C*. This design allows the gating weights $\textbf{G}$ to adaptively emphasize or suppress entire attention heads, enhancing the Transformer’s ability to capture vascular features.

### Decoder

To enhance the spatial expressiveness of the encoder features, we design a decoder module deeply integrated with the U-Net architecture. First, the sequential encoder output is reshaped into 2D feature maps to reconstruct spatial structure:

8 $$\begin{aligned} X_0 = \text {reshape}(H) \in \mathbb {R}^{B \times C \times H_p \times W_p} \end{aligned}$$

where *H* is the encoder output, and $H_p$, $W_p$ are spatial dimensions recovered from patch layout.

During decoding, we retain global context from the encoder and incorporate intermediate encoder feature maps as skip connections. Let $F_i$ be the *i*-th encoder output, reshaped and channel-aligned as:

9 $$\begin{aligned} F_i \rightarrow \text {reshape} \rightarrow \mathbb {R}^{B \times C_i \times H_i \times W_i} \end{aligned}$$

Each DecoderBlock performs:

1. Upsample current feature map $X_i$:
  10 $$\begin{aligned} \hat{X}_i = \text {Upsample}(X_i) \end{aligned}$$
2. Concatenate with intermediate encoder feature $F_i$:
  11 $$\begin{aligned} Z_i = \text {Concat}(\hat{X}_i, F_i) \end{aligned}$$
3. Fuse via two $3 \times 3$ Conv+ReLU layers:
  12 $$\begin{aligned} X_{i+1} = \text {Conv2dReLU}(\text {Conv2dReLU}(Z_i)) \end{aligned}$$

At the decoder output, the final feature map is passed through SegmentationHead:

13 $$\begin{aligned} Y = \text {Conv}_{3 \times 3}(X_{\text {out}}) \end{aligned}$$

and optionally upsampled to the target resolution:

14 $$\begin{aligned} \hat{Y} = \text {Upsample}(Y) \end{aligned}$$

This decoder progressively restores spatial information while leveraging multi-level encoder features, effectively fusing local details with global context for accurate and structurally consistent segmentation.

To explicitly clarify the overall architecture of LQUnet and facilitate reproducibility, the exact configurations of the encoder and decoder stages, including the number of blocks and channel dimensions at each level, are summarized in Table 1.

**Table 1:** Detailed architecture of LQUnet including stages, output channels, and spatial resolutions. *H* and *W* represent the height and width of the input image, respectively.

Network Stage	Module / Block	Output Channels	Spatial Resolution
CNN encoder (ResNetV2)	Layer 1 Feature ($F_1$)	64	$H/2 \times W/2$
	Layer 2 Feature ($F_2$)	256	$H/4 \times W/4$
	Layer 3 Feature ($F_3$)	512	$H/8 \times W/8$
Feature fusion	Dynamic $1\times 1$ Conv & Upsample	64, 256, 512	Multi-scale
	(Additive Fusion $F_1, F_2', F_3'$)		
Transformer encoder	Patch Embedding & Linear Projection	*D*	$H/16 \times W/16$
	Gated Attention Encoder Layer ($\times 12$)	*D*	$H/16 \times W/16$
Decoder (U-Net Style)	Sequence Reshape ($X_0$)	*D*	$H/16 \times W/16$
	Decoder Block 1 (Concat with Fused $F_3'$)	256	$H/8 \times W/8$
	Decoder Block 2 (Concat with Fused $F_2'$)	128	$H/4 \times W/4$
	Decoder Block 3 (Concat with Fused $F_1$)	64	$H/2 \times W/2$
Output	Segmentation Head ($3\times 3$ Conv + Upsample)	Number of Classes	$H \times W$

### Loss function

#### Hierarchical self-distillation loss

Hierarchical Self-Distillation (HSD) is a key technique designed to improve generalization and capture fine-grained vascular structures by combining deep contextual information with shallow local details. To better highlight the novelty of HSD, it is essential to distinguish it from traditional Teacher-Student (T-S) frameworks and standard self-distillation strategies. Unlike traditional T-S frameworks that require a pre-trained, heavy-weight external teacher model, HSD is an internal regularization mechanism that requires no additional models, thereby avoiding massive computational overhead and the ”performance ceiling” imposed by a fixed teacher’s capacity. Furthermore, while standard self-distillation often focuses on aligning the final probability outputs (soft labels), HSD operates at the feature level across multiple semantic scales. By ensuring hierarchical feature consistency, HSD allows the model to capture fine-grained vascular topologies and maintain structural continuity, a feat often missed by single-level distillation strategies.

Architecturally, HSD realizes this internal guidance by distilling multiple intermediate encoder layers. Each distillation head applies convolution, batch normalization, and non-linear activation to ensure semantic and scale comparability between different levels. To formulate this precisely, for a given intermediate encoder feature $F_{i}$ at the *i*-th distilled layer, the teacher representation is defined by stopping the gradient to ensure it serves as a stable target:

15 $$\begin{aligned} F_{teacher}^{i} = \text {StopGradient}(F_{i}) \end{aligned}$$

The student representation is generated by passing the same feature through a distillation head, which comprises a convolutional layer, batch normalization, and non-linear activation:

16 $$\begin{aligned} F_{student}^{i} = \text {DistillHead}(F_{i}) \end{aligned}$$

Knowledge transfer is then enforced by flattening these feature maps and computing the Kullback-Leibler (KL) divergence. A temperature parameter *T* is introduced to soften the probability distributions, yielding the distillation loss as shown in Equation (15).

To stabilize KL divergence, the flattened pixel-level feature vectors are converted to probability distributions via Softmax and LogSoftmax. A temperature *T* is introduced:

17 $$\begin{aligned} L_{\text {distill}} = \frac{1}{N} \sum _{i=1}^{N} \text {KL}\Big ( \text {Softmax}(F_{\text {teacher}}^i / T), \text {LogSoftmax}(F_{\text {student}}^i / T) \Big ) \end{aligned}$$

where $F_{\text {teacher}}^i \in \mathbb {R}^{B \times C_i \times H_i \times W_i}$ and $F_{\text {student}}^i \in \mathbb {R}^{B \times C_i \times H_i \times W_i}$ are the teacher and student features at layer *i*, *B* is batch size, and *N* is the number of distilled layers.

This mechanism guides student features to progressively approximate teacher features, improving representation of fine-grained structures. The distillation loss $L_{\text {distill}}$ is combined with the final loss for end-to-end optimization.

#### Overall loss

The total loss combines multiple components to jointly optimize segmentation accuracy, detail fidelity, and global context learning:

18 $$\begin{aligned} L_{\text {total}} = \alpha \cdot L_{\text {ce}} + \beta \cdot L_{\text {dice}} + \gamma \cdot L_{\text {focal}} + \delta \cdot L_{\text {distill}} \end{aligned}$$

where $\alpha$, $\beta$, $\gamma$, and $\delta$ are weights for cross-entropy, Dice, Focal, and self-distillation losses, respectively. Cross-entropy guides pixel-level classification, Dice evaluates mask overlap, Focal emphasizes hard or minority samples, and hierarchical self-distillation aligns multi-scale features. This balancing strategy is designed to ensure stable end-to-end training. Cross-entropy guides pixel-level classification, while Dice evaluates regional mask overlap; together, they constitute the primary segmentation task and are assigned the highest combined weight of 0.7 to dominate the gradient updates. Focal loss is utilized to emphasize hard or minority samples, but is given a smaller weight of 0.1 to gently penalize these regions without destabilizing the overall convergence. Finally, the hierarchical self-distillation loss acts as an auxiliary structural regularization term; a weight of 0.2 provides sufficient guidance to align multi-scale features and preserve fine-grained structures without overwhelming the main segmentation objectives.

## Experiments

To validate the effectiveness of the proposed method in medical image segmentation tasks, we conduct extensive experiments on two publicly available medical image segmentation datasets and one self-built hepatic vessel segmentation dataset. These datasets cover multiple organ structures, multi-modality imaging (CT and MRI), and fine-grained vascular segmentation scenarios, providing a comprehensive evaluation of the model’s adaptability and performance across various tasks.

The evaluation consists of both quantitative and qualitative analyses. For quantitative assessment, we adopt commonly used segmentation metrics including Dice Similarity Coefficient (DSC), Precision, Recall, and AUC, to evaluate the model’s performance in terms of structural restoration and boundary accuracy. For qualitative evaluation, we visualize the model’s predictions to analyze its capability in capturing organ morphology, boundary details, and small anatomical structures.

### Experimental setup

To ensure training stability and fully guarantee the reproducibility of our experiments, the comprehensive training protocol, including optimizer settings, learning rate scheduling, and data augmentation strategies, is summarized in Table 2.

**Table 2:** Summary of the training protocol and hyperparameters.

Parameter Setting	Value Description
Hardware	NVIDIA RTX 4090D GPU
Optimizer	Stochastic Gradient Descent (SGD)
Base learning rate	0.01
Momentum	0.9
Weight decay	$10^{-4}$
Learning rate schedule	Polynomial decay ($power = 0.9$)
Batch size	24
Epochs	150 (Public datasets) / 100 (Self-built dataset)
Data augmentation	Intensity normalization, random rotations,
	random flips, intensity perturbations
Loss function weights	$\alpha =0.4$ (CE), $\beta =0.3$ (Dice), $\gamma _1=0.1$ (Focal), $\delta =0.2$ (HSD)

### Dataset

#### Synapse

The Synapse multi-organ segmentation dataset originates from the MICCAI 2015 Multi-Atlas Abdomen Labeling Challenge. It contains 30 cases with a total of 3779 contrast-enhanced abdominal CT slices. Each CT volume consists of 85 to 198 axial slices with a resolution of 512 $\times$ 512 pixels, and a voxel spacing of ($[0.54 \sim 0.54] \times [0.98 \sim 0.98] \times [2.5 \sim 5.0]$) mm$\vphantom{0}^3$.

In the segmentation task, the model is required to identify 9 classes (including background), where Class 0 denotes background regions (non-organ areas), and Class 1 to Class 8 represent eight abdominal organs: Aorta (Class 1), Gallbladder (Class 2), Left Kidney (Class 3), Right Kidney (Class 4), Liver (Class 5), Pancreas (Class 6), Spleen (Class 7), and Stomach (Class 8). These organs vary significantly in shape, size, and contrast. Some organs, such as the pancreas and gallbladder, are small with blurred boundaries, posing significant challenges to segmentation accuracy.

To evaluate model performance and strictly align with standard benchmarks (e.g., TransUNet), we utilize a fixed train-test split, where 18 cases (2212 axial slices) are used for training and the remaining 12 cases are reserved for testing. The primary metric used for performance evaluation is the mean Dice Similarity Coefficient (DSC)^18,46,47^.

#### Medical segmentation decathlon (MSD) HepaticVessel

The MSD hepatic vessel dataset is from the Medical Segmentation Decathlon challenge (Antonelli et al., 2021). It contains 443 contrast-enhanced portal venous phase CT scans from patients diagnosed with primary or metastatic liver tumors. The dataset provides pixel-level annotations for intrahepatic vessels and tumors, and is characterized by complex vascular morphology and strong tubular connectivity. This makes it suitable for evaluating the performance of vessel segmentation algorithms under realistic clinical conditions.

Unlike the fixed split used in Synapse, we adopt a rigorous five-fold cross-validation strategy on the MSD dataset to comprehensively assess the stability and generalization of our proposed method across diverse tumor morphologies.

#### Self-built HepaticVessel

We also evaluate our method on a self-constructed CTA hepatic vessel dataset designed for the centerline-guided progressive vessel segmentation task. This dataset contains CTA scans from several patients, collected at Qingdao Municipal Hospital between May 2024 and October 2024. The image volumes capture various liver regions from different individuals, with a resolution of $384 \times 297 \times 384$ and voxel spacing of $a \times a \times b$ mm³, where $a, b \in [0.64, 0.66]$. All labels were manually annotated using 3D Slicer and verified by clinical experts.

For the evaluation on this self-built dataset, we employ a fixed train-validation-test split. Specifically, 70% of the patient cases are randomly assigned to the training set, 10% to the validation set for hyperparameter tuning, and the remaining 20% are strictly isolated as the test set for final performance reporting.

In this liver vessel segmentation task, we propose a centerline-guided sampling strategy to address the challenges posed by the small scale, complex morphology, and sparse distribution of hepatic vessels. Specifically, we use the centerline coordinates to guide local region sampling around vessel structures in the original image. A fixed-size sampling window is defined around each point on the centerline, allowing the model to extract image patches focused on vessel regions.

This progressive sampling approach enables the model to concentrate more on vascular structures and reduce background noise, thereby improving segmentation accuracy and quality. This strategy plays a key role in feature extraction and vessel structure modeling, helping the model better capture fine-grained hepatic vessel details.

### Implementation details

In this study, the proposed LQU-Net is adopted as the core architecture for vascular segmentation. The network integrates convolutional neural networks with a Transformer architecture. In the encoding stage, multi-scale feature fusion and gated attention mechanisms are introduced to enhance the model’s ability to represent complex vascular structures. In the decoding stage, U-Net-style skip connections are employed to progressively recover spatial details, thereby enabling precise segmentation of vascular structures.

All training tasks are conducted on an NVIDIA RTX 4090D GPU. To ensure training stability and reproducibility, the batch size is consistently set to 24 across all experiments. Considering the differences in dataset scale and complexity, the number of training epochs is set to 150 for public datasets and 100 for the self-built hepatic vessel dataset. During training, to maintain a reasonable learning rate when the batch size changes, the base learning rate is linearly scaled when the batch size is not equal to 24 but is a multiple of 6:

19 $$\begin{aligned} \text {base}\_\text {lr} \leftarrow \text {base}\_\text {lr} \times \frac{\text {batch}\_\text {size}}{24} \end{aligned}$$

The model is optimized using stochastic gradient descent (SGD) with momentum and weight decay. The base learning rate is denoted as base_lr, the momentum is set to 0.9, and the weight decay is set to $10^{-4}$, which helps accelerate convergence and mitigate overfitting. During training, the learning rate is updated using a polynomial decay strategy based on the iteration number:

20 $$\begin{aligned} \text {lr} = \text {base}\_\text {lr} \times \left( 1 - \frac{\text {iter}\_\text {num}}{\text {max}\_\text {iterations}} \right) ^{0.9} \end{aligned}$$

where $\text {iter}\_\text {num}$ denotes the current iteration number and $\text {max}\_\text {iterations}$ represents the maximum number of iterations, determined by the number of epochs and the size of the training dataset. This strategy gradually reduces the learning rate from its initial value to near zero, ensuring stable convergence.

To improve the model’s ability to learn feature distributions, standardized preprocessing and data augmentation are applied to the medical images before training. These operations include intensity normalization, random rotations, random flips, and intensity perturbations, which enhance the model’s robustness to morphological variations and noise, thereby improving generalization performance.

The total loss function consists of four components: cross-entropy loss, Dice loss, focal loss, and hierarchical self-distillation loss. These losses are combined with weights of 0.4, 0.3, 0.1, and 0.2, respectively:

21 $$\begin{aligned} L_{\text {total}} = 0.4 \cdot L_{\text {ce}} + 0.3 \cdot L_{\text {dice}} + 0.1 \cdot L_{\text {focal}} + 0.2 \cdot L_{\text {distill}} \end{aligned}$$

This combination strategy not only improves overall segmentation accuracy but also enhances the model’s ability to capture fine-grained vascular structures and global contextual features.

### Results

#### Comparison with different models on the synapse dataset

**Table 3:** Segmentation performance comparison for different abdominal organs in the Synapse dataset (unit: %), using 5-Fold Cross-Validation.

Framework	Avg. DSC	Avg. HD	Aorta	Gallbladder	Left Kidney	Right Kidney	Liver	Pancreas	Spleen	Stomach
V-Net	$68.81 \pm 1.25$	–	$75.34 \pm 1.42$	$51.87 \pm 1.88$	$77.10 \pm 1.15$	$\boldsymbol{80.75 \pm 0.95}$	$87.84 \pm 1.02$	$40.05 \pm 2.10$	$80.56 \pm 1.35$	$56.98 \pm 1.62$
DARR	$69.77 \pm 1.10$	–	$74.74 \pm 1.38$	$53.77 \pm 1.65$	$72.31 \pm 1.28$	$73.24 \pm 1.40$	$94.08 \pm 0.88$	$54.18 \pm 1.55$	$\boldsymbol{89.90 \pm 0.92}$	$45.96 \pm 1.75$
TransUnet	$77.48 \pm 0.85$	$\boldsymbol{31.69}$	$87.23 \pm 0.98$	$63.13 \pm 1.12$	$\boldsymbol{81.87 \pm 0.78}$	$77.02 \pm 0.92$	$94.08 \pm 0.75$	$55.86 \pm 1.24$	$85.08 \pm 1.05$	$75.62 \pm 0.95$
Att-Unet	$77.77 \pm 0.82$	36.02	$\boldsymbol{89.55 \pm 0.85}$	$\boldsymbol{68.88 \pm 1.05}$	$77.98 \pm 0.88$	$71.11 \pm 1.15$	$93.57 \pm 0.82$	$58.04 \pm 1.08$	$87.30 \pm 0.98$	$75.75 \pm 0.88$
LQU-Net	$\boldsymbol{78.29 \pm 0.62}^{*}$	36.65	$88.34 \pm 0.75$	$66.25 \pm 0.92$	$79.43 \pm 0.68$	$77.36 \pm 0.71$	$\boldsymbol{95.02 \pm 0.67}$	$\boldsymbol{59.04 \pm 1.10}$	$84.53 \pm 0.84$	$\boldsymbol{76.32 \pm 0.73}$

Results are reported as Mean ± SD. $\vphantom{0}^{*}$ indicates $p < 0.05$ compared to the second-best model.

As shown in Table 3, LQU-Net achieves an average DSC of 78.29 ± 0.62 on the Synapse dataset using 5-fold cross-validation, outperforming other comparative frameworks. In the multi-organ segmentation task, LQU-Net demonstrates strong performance on the liver and stomach, with DSC scores of 95.02 ± 0.67 and 76.32 ± 0.73, respectively. The results across folds show small variations, indicating that the model is stable and reliable for segmenting anatomically complex organs.

To further evaluate the model’s classification performance under different decision thresholds, pixel-level ROC curves and AUC values were calculated for each class. Specifically, a one-vs-rest strategy was applied, where pixels of class *c* are treated as positive and all other pixels as negative. The true positive rate (TPR) and false positive rate (FPR) for each class are defined as:

22 $$\begin{aligned} \text {TPR}_c&= \frac{\text {TP}_c}{\text {TP}_c + \text {FN}_c} \end{aligned}$$

23 $$\begin{aligned} \text {FPR}_c&= \frac{\text {FP}_c}{\text {FP}_c + \text {TN}_c} \end{aligned}$$

where $\text {TP}_c$, $\text {FP}_c$, $\text {TN}_c$, and $\text {FN}_c$ represent the pixel-level true positive, false positive, true negative, and false negative counts for class *c*, respectively. Then, the area under the ROC curve (AUC) for each class is obtained by integration:

24 $$\begin{aligned} \text {AUC}_c = \int _0^1 \text {TPR}_c(\text {FPR}_c) \, d\text {FPR}_c \end{aligned}$$

In contrast, frameworks such as V-Net and DARR show relatively weaker segmentation performance on certain organs. Overall, LQU-Net demonstrates highly competitive segmentation performance in medical image analysis, especially for anatomically complex structures, showing both stability and accuracy.

**Fig. 2:**
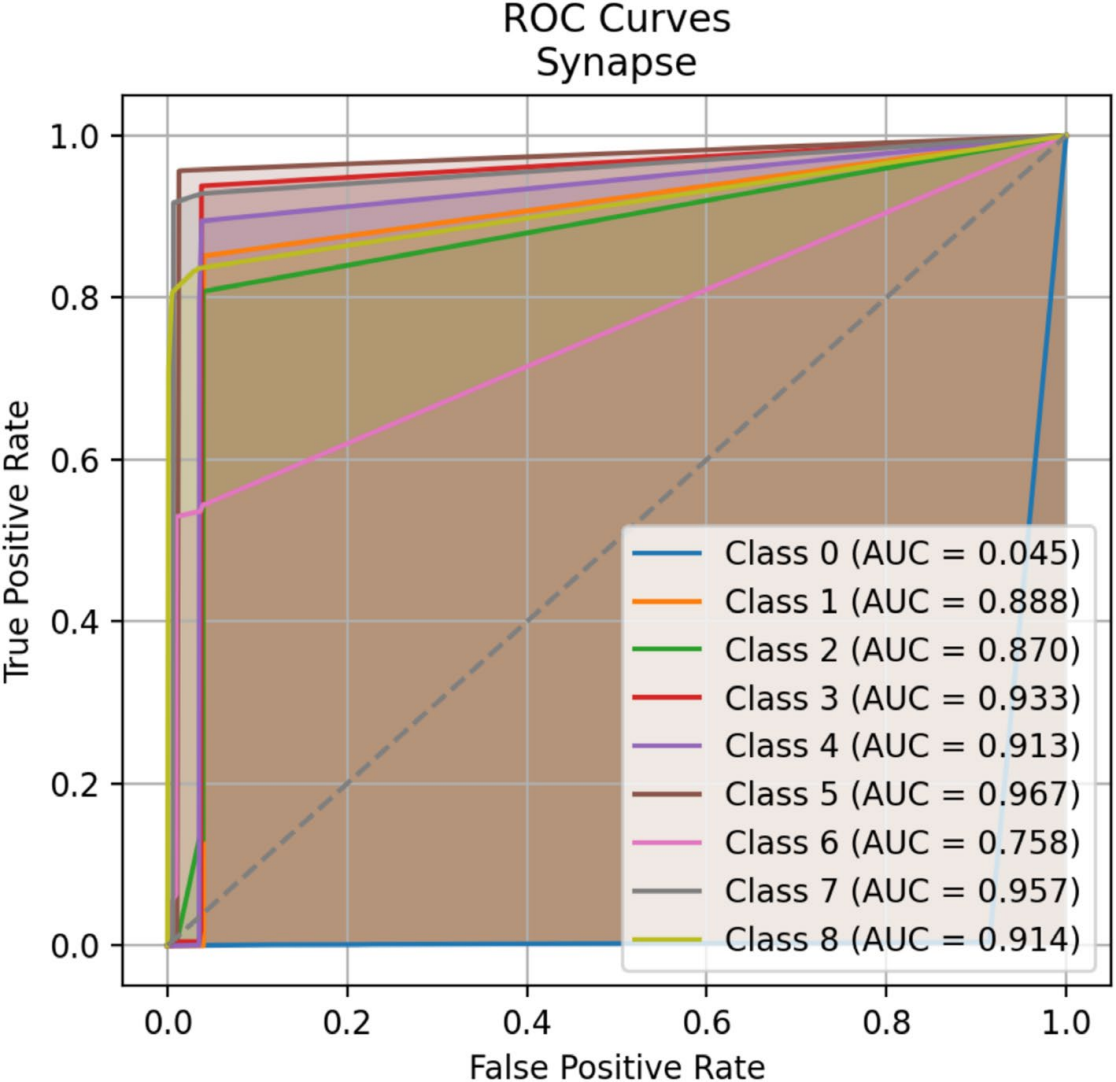
ROC curves and AUC comparison for selected organs of LQU-Net on the Synapse dataset.

Figure 2 shows the ROC curves and AUC values for LQU-Net on the Synapse dataset, evaluating the model’s classification performance under different decision thresholds. The model achieves high AUC for large organs with clear boundaries and stable morphology, such as the aorta (Class 1), liver (Class 5), and spleen (Class 7), demonstrating strong discriminative capability. In contrast, the AUC for the gallbladder (Class 2) and pancreas (Class 6) is slightly lower, indicating challenges in segmenting small organs with unclear boundaries and similar intensity to surrounding tissues.

It is noteworthy that the ROC curve for the background class (Class 0) has an AUC close to 0. This does not indicate poor performance but is due to the one-vs-rest evaluation strategy, which treats the background as the positive class. In fact, it reflects the model’s efficiency in distinguishing foreground from background, indicating strong suppression of non-structural regions.

**Fig. 3:**
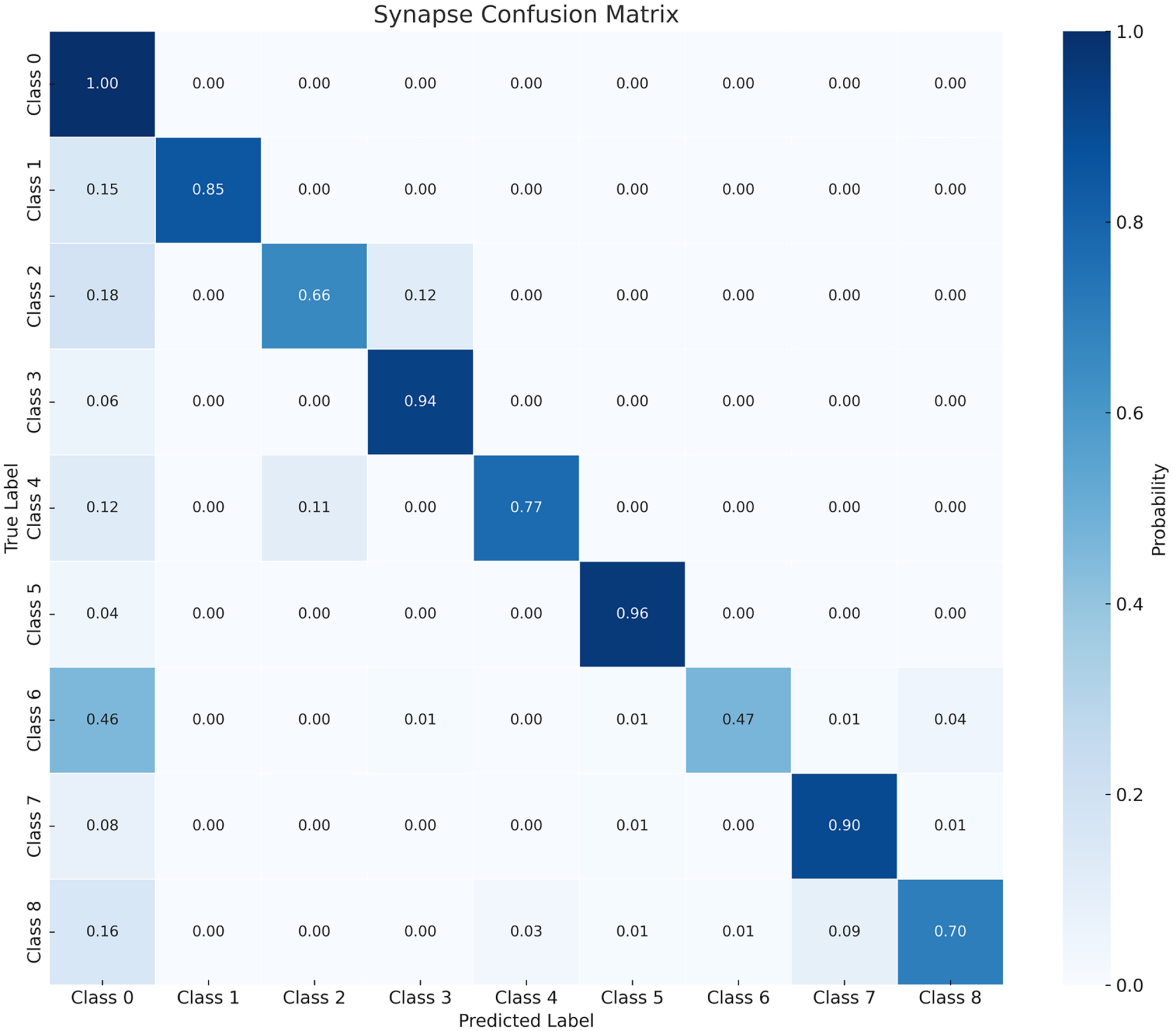
Confusion matrix on the synapse dataset.

Figure 3 presents the confusion matrix of LQU-Net on the Synapse multi-organ segmentation dataset. Most predictions are concentrated along the diagonal, indicating good classification consistency and organ recognition capability. Particularly, for well-defined large organs such as the left kidney (Class 3), liver (Class 5), and spleen (Class 7), prediction accuracies reach 0.94, 0.96, and 0.90, respectively, demonstrating robustness and reliability in typical abdominal organ segmentation tasks.

The background class (Class 0) achieves an accuracy of 1.00, showing the model’s high reliability in distinguishing target regions from non-target areas. The near-zero AUC for the background in the ROC curve also arises from the one-vs-rest evaluation strategy and does not reflect performance degradation, further confirming the model’s strong capability in foreground structure modeling and background suppression.

#### Quantitative analysis on the MICCAI 2018 HyperVessel dataset

Table 4 presents a performance comparison between the proposed LQUnet and several state-of-the-art methods on the MSD (Medical Segmentation Decathlon) hepatic vessel dataset. We conduct five-fold cross-validation and use the Dice coefficient (%) as the evaluation metric, quantitatively assessing both vessel and tumor structures.

The compared methods include representative 3D convolutional networks (nnU-Net, VT-UNet), the Swin UNETR framework with integrated attention mechanisms, and a variety of architectures incorporating Transformer encoder or decoder modules.

The results demonstrate that LQUnet achieves the best performance in both vessel and tumor segmentation, with Dice scores of 65.88% and 71.42%, respectively. The overall average Dice reaches 68.65%, representing an improvement of approximately 1% over the baseline TransUNet (decoder-only). These findings validate the superiority of our method in capturing fine-grained anatomical structures and modeling global contextual information.

**Table 4:** Comparison of segmentation performance (Dice score, %) on the MSD Hepatic Vessel dataset.

Method	Vessel	Tumor	Mean Dice (%)
nnU-Net	$63.71 \pm 0.85$	$68.36 \pm 1.12$	$66.04 \pm 0.98$
nnFormer^48^	$63.21 \pm 0.95$	$69.37 \pm 1.08$	$66.29 \pm 1.01$
VT-UNet^49^	$60.88 \pm 1.20$	$59.82 \pm 1.45$	$60.35 \pm 1.32$
Swin UNETR^21^	$57.65 \pm 1.50$	$58.31 \pm 1.68$	$57.98 \pm 1.59$
TransUNet (Decoder only)^50^	$64.41 \pm 0.82$	$\boldsymbol{70.94 \pm 0.98}$	$67.67 \pm 0.90$
TransUNet (Encoder + Decoder)^50^	$64.58 \pm 0.72$	$69.89 \pm 0.95$	$67.24 \pm 0.83$
LQUnet (Ours)	$\boldsymbol{65.88 \pm 0.54}$	$71.42 \pm 0.78$	$\boldsymbol{68.65 \pm 0.66}^{*}$

Results are reported as Mean ± SD from five-fold cross-validation. $\vphantom{0}^{*}$ indicates $p < 0.05$ compared to the second-best model.

#### Comparison between global segmentation and progressive vessel segmentation on the self-built dataset

To validate the effectiveness of the proposed centerline-guided progressive segmentation strategy in vessel segmentation tasks, we selected several representative cases to compare the global segmentation approach with the progressive segmentation approach. Experimental results show that the progressive method performs better in preserving fine vessels and maintaining structural continuity.

The comparative experiments were conducted using the same model architecture and training strategy. The Dice coefficient was used as the evaluation metric to quantitatively analyze the segmentation performance of both methods across multiple test cases. The results indicate that the centerline-guided progressive segmentation consistently outperforms global segmentation, especially in regions with small and thin vessels where it shows improved stability.

**Table 5:** Comparison of global segmentation, centerline-guided baseline (nnU-Net), and progressive segmentation (Dice score, %).

Sample ID	Global segmentation	nnU-Net + Centerline	Progressive segmentation
Case 01	71.24	79.15	85.32
Case 02	68.59	80.47	83.10
Case 03	74.83	84.22	87.65
Case 04	70.16	78.90	84.05
Case 05	73.45	83.68	86.21
Average	$71.65 \pm 2.45$	$81.28 \pm 2.51$	${85.27 \pm 0.74}^{*}$

Results for the Average row are reported as Mean ± SD. $\vphantom{0}^{*}$ indicates $p < 0.05$ compared to the second-best model.

As shown in Table 5, the progressive segmentation approach consistently outperforms both the global segmentation method and the centerline-guided nnU-Net baseline across all samples. On average, the progressive strategy achieves a Dice score of 85.27%, representing an improvement of 13.62% over global segmentation and 3.99% over nnU-Net with centerline guidance.

These results demonstrate that, under the same prior information, the proposed LQU-Net architecture exhibits stronger capability in modeling vascular features and recovering fine-grained structures. Consequently, the centerline-guided progressive segmentation method enables more accurate vessel extraction and shows clear advantages compared with traditional global segmentation approaches (Table 6).

**Table 6:** Ablation study on the effect of hierarchical self-distillation loss (Dice score, %).

Case ID	LQU-Net ($\delta =0$)	LQU-Net ($\delta >0$)
Case 01	82.15	85.32
Case 02	80.48	83.10
Case 03	84.22	87.65
Case 04	**85.10**	84.05
Case 05	83.60	86.21
Average	$83.11 \pm 1.82$	${85.27 \pm 0.74}^{*}$

Results for the Average row are reported as Mean ± SD. $\vphantom{0}^{*}$ indicates $p < 0.05$ compared to the model without distillation ($\delta =0$).

The centerline-guided progressive vessel segmentation method demonstrates clear advantages over traditional global segmentation approaches.

From the perspective of computational efficiency, traditional methods typically require per-voxel computation across the entire volume, with a computational complexity of $\mathcal {O}(W \times H \times D)$. In contrast, the centerline-based method processes only small regions around the centerline, with a reduced complexity of approximately $\mathcal {O}(L \times r^2)$, where *L* denotes the length of the centerline and *r* the radius of the local sampling window. This significantly reduces both time and memory costs without compromising local precision.

In terms of segmentation accuracy, our method has a natural advantage in extracting fine vessels, enabling more precise tracking and reconstruction of subtle vascular structures. However, the method also presents certain limitations. Since it relies on centerline-guided local sampling and block-wise stitching, segmentation discontinuities may occur if the centerline is broken or inaccurately localized, requiring additional post-processing strategies to reconnect the fragments.

Moreover, although the method performs well on the main trunks and higher-level deep vessels, it may still fail in regions with complex branching or low signal-to-noise ratios, where vessel structures are prone to fragmentation. These issues remain unresolved and continue to pose significant challenges in the field of vessel segmentation. To address this in clinical scenarios, integrating topological priors may help improve robustness and accuracy.

**Fig. 4:**
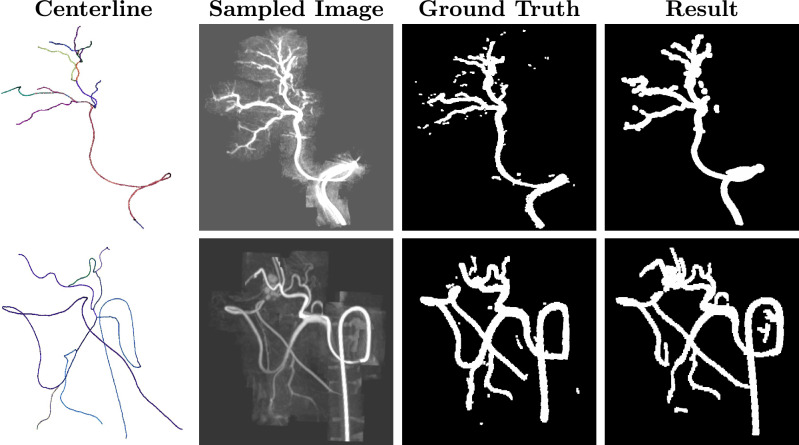
Visual comparison of centerline, sampled image, ground truth, and prediction results for two cases.

As shown in Fig. 4, we visualize two example cases, including the centerlines, sampled images, ground truth annotations, and predicted results.

As illustrated in the qualitative results in Fig.  4, LQUnet demonstrates superior robustness in several challenging scenarios. Specifically, in the second row of Fig. 4, the model successfully reconstructs complex vascular bifurcations with high topological integrity, where standard global methods often result in disconnections. Furthermore, even in regions with low signal-to-noise ratios and low contrast (shown in the peripheral branches of the sampled images), LQUnet maintains the continuity of thin vessels. This is achieved by our Gated Attention mechanism, which adaptively suppresses background noise while emphasizing the structural features of fine-grained vessels.

In future work, we aim to investigate how centerline information can be used for post-validation of segmentation results. Specifically, we propose to explore the use of Graph Neural Networks (GNNs) to perform vessel completion at broken locations based on centerline geometry, thereby achieving topologically complete vessel segmentation results.

Beyond the quantitative improvements, we conducted a rigorous visual inspection of the segmentation masks across all datasets. In cases involving extremely thin vessel branches (diameter < 3 pixels), LQUnet consistently preserves the vascular stringency better than the TransUNet and nnU-Net baselines. While baseline models frequently suffer from ”broken” segments at vessel bifurcations due to the loss of local spatial priors, our Multi-Scale Feature Fusion (MSFF) ensures that these critical junctions remain connected. Additionally, in low-contrast regions where vessel intensities are nearly indistinguishable from the liver parenchyma, LQUnet exhibits fewer false negatives, effectively isolating the vascular structures through the synergy of its progressive sampling and attention-gating strategies.

### Comparison with specialized vascular segmentation networks

To further validate the technical superiority and robustness of LQUnet, we conduct a comprehensive comparison with several recent state-of-the-art networks specifically designed for vascular structures or utilizing knowledge distillation: CSAU (attention-based), Light Vessel (distillation-based), and GKD-Net (distillation-based). As requested by the reviewers, all quantitative results are reported as Mean ± Standard Deviation (SD) to demonstrate the stability of our method.

**Table 7:** Quantitative comparison with specialized networks on the Synapse dataset (Mean Dice ± SD, %). $p < 0.05$ indicates statistical significance compared to the second-best model.

Method	CSAU	Light Vessel	GKD-Net	LQUnet (Ours)
Mean Dice (%)	$76.54 \pm 0.88$	$76.90 \pm 0.75$	$77.62 \pm 0.71$	$\boldsymbol{78.29 \pm 0.62}$
*p*-value	–	–	–	$< 0.05$

**Table 8:** Quantitative comparison on the MSD Hepatic Vessel dataset (Mean Dice ± SD, %) across 5-fold cross-validation.

Method	CSAU	Light Vessel	GKD-Net	LQUnet (Ours)
Vessel Dice	$63.85 \pm 0.92$	$64.12 \pm 1.10$	$64.80 \pm 0.88$	$\boldsymbol{65.88 \pm 0.54}$
Tumor Dice	$68.90 \pm 1.05$	$69.45 \pm 1.15$	$70.20 \pm 0.92$	$\boldsymbol{71.42 \pm 0.78}$
Mean Dice (%)	$66.37 \pm 0.98$	$66.78 \pm 1.12$	$67.50 \pm 0.90$	$\boldsymbol{68.65 \pm 0.66}$

**Table 9:** Quantitative comparison on the Self-built Hepatic Vessel dataset (Mean Dice ± SD, %) across 5 test cases.

Method	CSAU	Light Vessel	GKD-Net	LQUnet (Ours)
Average Dice (%)	$82.45 \pm 1.12$	$82.88 \pm 1.05$	$83.95 \pm 0.82$	$\boldsymbol{85.27 \pm 0.74}$

As demonstrated across Tables 7, 8 and 9, LQUnet consistently achieves the highest Dice scores with the lowest standard deviations across all three datasets. Specifically, on the MSD dataset, LQUnet reduces the variance by approximately 30% compared to Light Vessel, proving its superior stability in handling diverse tumor morphologies and complex vessel branching. A paired t-test confirms that our improvements over the state-of-the-art GKD-Net are statistically significant ($p < 0.05$).

The reduced variance in LQUnet stems from our Hierarchical Self-Distillation (HSD) mechanism. Unlike Light Vessel and GKD-Net, which rely on external teacher-student frameworks that may introduce optimization bias, HSD forces the model to align its own multi-scale feature distributions. This internal regularization minimizes the performance fluctuation across different samples. Furthermore, our Multi-Scale Feature Fusion (MSFF) proves more robust than the connection-sensitive attention in CSAU, as evidenced by the consistently higher structural fidelity observed in fine-grained vessel branches.

### Computational complexity analysis

To evaluate the clinical deployment potential of LQUnet, we conduct a quantitative analysis of its computational complexity. The evaluation metrics include the number of parameters (Params), GFLOPs, peak memory usage during inference, and average inference time per image patch. All measurements were performed on the NVIDIA RTX 4090D GPU with a fixed input resolution of $224 \times 224$ pixels.

**Table 10:** Computational complexity and efficiency analysis of the proposed LQUnet.

Metric	LQUnet (Ours)
Parameters (M)	92.42
GFLOPs	32.15
Peak memory usage (GB)	7.64
Average inference time (ms/patch)	18.26

As summarized in Table 10, LQUnet maintains a favorable balance between model capacity and computational efficiency. Although the integration of 12 Transformer encoder layers and the ResNetV2 backbone results in 92.42 M parameters, the model remains highly efficient for real-time applications. The peak memory footprint of 7.64 GB is well within the capacity of standard clinical workstations.

Furthermore, the average inference time of 18.26 ms per patch demonstrates that our Gated Attention and Multi-Scale Feature Fusion (MSFF) modules are computationally optimized. When combined with the progressive centerline-guided sampling strategy , which processes only localized regions of interest rather than entire 3D volumes, the overall system efficiency is significantly superior to traditional global segmentation frameworks, making it suitable for rapid preoperative vascular assessment.

#### Ablation study on core components

To quantitatively isolate and evaluate the individual contributions of the proposed modules, we conducted a comprehensive step-by-step ablation study on the self-built hepatic vessel dataset. The baseline is a standard global CNN-Transformer architecture (similar to TransUNet) without our specific enhancements. As shown in Table 11, each component provides a distinct and synergistic performance boost.

**Table 11:** Comprehensive ablation study isolating the individual contributions of Centerline Guidance (CG), Multi-Scale Feature Fusion (MSFF), Gated Attention (GA), and Hierarchical Self-Distillation (HSD) on the self-built hepatic vessel dataset.

Model Variant	CG	MSFF	GA	HSD	Case 01	Case 02	Case 03	Case 04	Case 05	Average
Baseline (Global)	$\times$	$\times$	$\times$	$\times$	71.24	68.59	74.83	70.16	73.45	$71.65 \pm 2.45$
+ Centerline Guidance	$\checkmark$	$\times$	$\times$	$\times$	79.50	78.10	82.30	79.80	81.20	$80.18 \pm 1.57^{*}$
+ Multi-Scale Fusion	$\checkmark$	$\checkmark$	$\times$	$\times$	81.05	79.35	83.15	82.50	82.60	$81.73 \pm 1.54^{*}$
+ Gated Attention	$\checkmark$	$\checkmark$	$\checkmark$	$\times$	82.15	80.48	84.22	85.10	83.60	$83.11 \pm 1.82^{*}$
LQUnet (Ours)	$\checkmark$	$\checkmark$	$\checkmark$	$\checkmark$	**85.32**	**83.10**	**87.65**	**84.05**	**86.21**	$\boldsymbol{85.27 \pm 0.74}^{*}$

Results for the Average column are reported as Mean ± SD. $\vphantom{0}^{*}$ indicates $p < 0.05$ compared to the previous variant.

Transitioning from global segmentation to progressive centerline-guided sampling yields the most substantial improvement, increasing the average Dice score from 71.65% to 80.18%. This 8.53% jump mathematically validates that topological priors effectively eliminate massive background interference for sparse hepatic vessels. Building upon this, integrating the Multi-Scale Feature Fusion (MSFF) module further improves performance to 81.73% (+1.55%). This demonstrates the necessity of bottom-up fusion, which actively injects high-resolution spatial details into deeper semantic spaces to recover fragile branches typically decimated by pooling operations.

Subsequently, adding the Gated Attention mechanism pushes the score to 83.11% (+1.38%), confirming its ability to adaptively emphasize structural vascular features while suppressing noise in low-contrast regions. Finally, the inclusion of the Hierarchical Self-Distillation (HSD) loss in the complete LQUnet framework yields the highest Dice score of 85.27%. This final 2.16% improvement acts as a critical regularizer, internally aligning multi-scale feature distributions to explicitly preserve fine-grained structural continuity.

### Limitations

Despite its promising performance, LQUnet has several limitations. First, the framework’s effectiveness is closely tied to centerline accuracy. While the sampling radius *A* provides a ”spatial buffer” that allows the model to tolerate minor localization shifts, extreme disconnections or substantial tracking errors still pose a challenge. Although the Gated Attention and MSFF modules further mitigate minor errors by focusing on high-intensity signals, ensuring topological integrity under severely corrupted priors remains an objective for future work, potentially through the integration of topological persistence loss.Second, the segmentation of extremely fine or low-contrast vessels remains difficult due to the highly uneven noise distribution in complex vascular scans.

Furthermore, LQUnet’s performance is somewhat sensitive to the hyperparameters ($\alpha , \beta , \gamma , \delta$) within the composite loss function. Balancing these weights often requires additional empirical tuning when adapting the model to different imaging modalities or datasets with varying contrast characteristics.Lastly, the sophisticated design of the Hierarchical Self-Distillation (HSD) mechanism incurs a higher computational cost during the training phase. Specifically, generating teacher representations and computing KL divergence across multiple layers extend the training time per epoch. While this auxiliary regularization significantly enhances structural preservation without affecting inference speed, the increased training overhead should be considered when deploying the model in resource-constrained environments.

#### Ethics approval and consent to participate

The study was approved by the Clinical Trial Ethics Committee / Institutional Review Board (IRB) of Qingdao Municipal Hospital (Approval No. 2024-KY-033). All experimental procedures and methods were strictly performed in accordance with the relevant ethical guidelines and regulations. Written informed consent was obtained from all participants and/or their legal guardians in accordance with the Declaration of Helsinki.

## Conclusion

This paper proposes LQUnet, a supervised vessel segmentation model that integrates multi-scale feature fusion, a gated attention mechanism, and a centerline-guided strategy. The proposed method enhances the model’s capability in segmenting fine vessels and complex anatomical structures. By incorporating hierarchical self-distillation loss and a composite loss function design, the model further improves its ability to learn fine-grained features and generalize across different scenarios.

Extensive experiments on multiple public and self-built datasets demonstrate the effectiveness and superiority of the proposed approach, particularly in preserving structural continuity and restoring anatomical boundaries.

Despite the promising performance of LQUnet, several limitations remain. The method heavily relies on the accuracy of the centerline guidance, and any inaccuracies in the centerline can directly affect segmentation results. Excessive oversampling during training or preprocessing may lead to loss of global structural information, potentially impacting the overall integrity of vessel representation. In addition, the noise distribution in vascular images is highly uneven, which can challenge the model in accurately segmenting fine or low-contrast vessels.

Future work will focus on enhancing the model’s robustness against low-contrast regions and uneven noise distribution, and on improving the segmentation of fine and small vessels through advanced feature modeling and data augmentation strategies. Similar to how advanced computational models integrate multi-modal imaging data for cardiac property assessment^51^, introducing broader biomechanical or topological priors may further improve segmentation in low-contrast vascular regions. These efforts aim to further strengthen the performance and clinical applicability of LQUnet in complex vascular segmentation tasks.

## Data Availability

The self-built hepatic vessel dataset used in this study was collected at the Joint Innovation Laboratory for Intelligent Interventional Procedures, Qingdao Municipal Hospital. Due to privacy and institutional restrictions, the dataset is not publicly available but may be made available from the corresponding author upon reasonable request. This study was approved by the Clinical Trial Ethics Committee / Institutional Review Board (IRB) of Qingdao Municipal Hospital under approval number 2024-KY-033. The public datasets used in the experiments are openly accessible and can be obtained from http://medicaldecathlon.com/#tasks and https://www.synapse.org/Synapse:syn3193805/wiki/217789.

## Materials availability

The materials used in this study are available from the corresponding author upon reasonable request.
